# Clinical and laboratorial outcome of different age-onset systemic lupus erythematosus patients in Jiangsu, China: a multicentre retrospective study

**DOI:** 10.1038/s41598-022-14840-4

**Published:** 2022-06-23

**Authors:** Lihui Wen, Ziyan Chen, Ziyi Jin, Wenyou Pan, Lin Liu, Min Wu, Fuwan Ding, Huaixia Hu, Xiang Ding, Hua Wei, Yaohong Zou, Xian Qian, Meimei Wang, Jian Wu, Juan Tao, Jun Tan, Zhanyun Da, Miaojia Zhang, Jing Li, Xuebing Feng, Jun Liang, Huayong Zhang, Lingyun Sun

**Affiliations:** 1 Department of Rheumatology and Immunology, Affiliated Nanjing Drum Tower Hospital, Medical School of Nanjing University, 321 Zhongshan Road, Nanjing, 210008 China; 2 Department of Rheumatology, Huai’an First People’s Hospital, Huai’an, China; 3 Department of Rheumatology, Xuzhou Central Hospital, Xuzhou, China; 4 Department of Rheumatology, Third Affiliated Hospital of Soochow University, Changzhou, China; 5 Department of Endocrinology, Yancheng Third People’s Hospital, Yancheng, China; 6 Department of Rheumatology, Lianyungang Second People’s Hospital, Lianyungang, China; 7 Department of Rheumatology, Lianyungang First People’s Hospital, Lianyungang, China; 8 Department of Rheumatology, Northern Jiangsu People’s Hospital, Yangzhou, China; 9 Department of Rheumatology, Wuxi People’s Hospital, Wuxi, China; 10 Department of Rheumatology, Jiangsu Province Hospital of Traditional Chinese Medicine, Nanjing, China; 11 Department of Rheumatology, Southeast University Zhongda Hospital, Nanjing, China; 12 Department of Rheumatology, First Affiliated Hospital of Soochow University, Suzhou, China; 13 Department of Rheumatology, Wuxi Hospital of Traditional Chinese Medicine, Wuxi, China; 14 Department of Rheumatology, Zhenjiang First People’s Hospital, Zhenjiang, China; 15 Department of Rheumatology, Affiliated Hospital of Nantong University, Nantong, China; 16 Department of Rheumatology, Jiangsu Province Hospital and Nanjing Medical University First Affiliated Hospital, Nanjing, China; 17 Department of Rheumatology, Affiliated Hospital of Jiangsu University, Zhenjiang, China

**Keywords:** Autoimmune diseases, Systemic lupus erythematosus, Autoimmunity

## Abstract

Studies on clinical features of systemic lupus erythematosus among different age-onset patients are lacking in China. This multicentre study aimed to systemically compare clinical manifestations, comorbidities, organ involvement, and laboratory findings among 797 Chinese juvenile-onset, adult-onset, and late-onset SLE (JSLE, ASLE, and LSLE) patients. They were classified into JSLE, ASLE, and LSLE groups if first diagnosed at < 18, 18–50, and > 50 years old, respectively. Chi-square test and analysis of variance were employed for categorical and continuous variables respectively. In younger-onset patients, the SLE Disease Activity Index 2000 score was significantly higher (JSLE vs. ASLE vs. LSLE = 17.43 ± 9.139 vs. 16.34 ± 8.163 vs. 14.08 ± 6.474, *p* = 0.031). Mucocutaneous symptoms (79.5% vs. 73.4% vs. 62.0%, *p* = 0.042), especially malar rash (76.1% vs. 66.1% vs. 53.5%, *p* = 0.011) occurred more frequently, and proteinuria rate was higher (54.5% vs. 56.3% vs. 36.6%, *p* = 0.007). In later-onset patients, cardiopulmonary involvement increased (11.4% vs. 24.3% vs. 29.6%, *p* = 0.012). In ASLE, hypoalbuminemia rate elevated (46.6% vs. 59.9% vs. 47.9%, *p* = 0.015). Our study demonstrated in a Chinese population that JSLE may be more active and suffer mucocutaneous disorders, while LSLE tended to suffer cardiopulmonary involvement at-onset. These findings may help identify treatment priorities when facing different age-onset SLE patients.

## Introduction

Systemic lupus erythematosus (SLE) is a systemic autoimmune disease affecting multiple organs and systems. Genetics, hormones, environment along with many other factors interact to trigger the breakdown of adaptive and innate immunity^1^. The clinical manifestations and autoantibody profiles of SLE are highly diverse, and this heterogeneity often causes confusion in clinical decision-making.

Although SLE mostly occurs in women during reproductive years, people of all ages can be patients. In years of clinical practice, we have observed a noticeable difference in clinical manifestations among patients of different ages at onset. In agreement with our findings, age-onset is considered to be a major factor associated with SLE clinical features^2^. Efforts have been made across the world to identify the association^3–10^. Studies from Spain, Portugal, Canada, Egypt, Korea, and other countries have demonstrated differences in disease activity, clinical manifestations, comorbidities, and morbidities in SLE patients of different age-onset^9,11–14^. However, no consensus has been reached so far. For example, in Spain, Portugal, Egypt, and Korea, disease activity was found to be higher in younger-onset patients, but in the Canadian population, adult-onset SLE was more active than childhood-onset SLE. The most common symptoms identified in each age-onset group were also not consistent among studies. The inconsistency might be caused by the ethnic variations of the study population. The differences in clinical features of different age-onset SLE patients deserve more attention from rheumatologists.

China has a large population of SLE patients, but a systemic comparison of clinical features among three age-onset groups is lacking. In order to get a better understanding of the relationship between age-onset and clinical features of SLE patients in China, we hereby compared detailed clinical manifestations, comorbidities, organ involvement, and laboratory findings among juvenile-onset, adult-onset, and late-onset SLE patients.

## Results

Of 797 SLE patients investigated, most (80.0%) were adult-onset. No significant gender differences occurred among groups, with women accounting for over 90% of each population.

The top three clinical manifestations at diagnosis were renal disorder (87.6%), malar rash (66.5%), and arthritis (66.1%), respectively. For the JSLE group, a significantly higher incidence of malar rash (76.1%, *p* = 0.011) was observed. The renal dysfunction affected approximately 90% of patients in the JSLE group. Although not significant, the LSLE group tends to suffer more often from serositis (23.9% vs. 19.7%, *p* = 0.234). No specific clinical patterns were found in the ASLE group compared to the total population.

The mean SLE Disease Activity Index 2000 (SLEDAI-2 K) score indicating disease activity was 16.26 ± 8.168 on initial admission, being the highest in the JSLE group (17.43 ± 9.139) and decreasing significantly with age (*p* = 0.031) (Table 1).

**Table 1:** Basic clinical features of patients from each age-onset group.

	All (n = 797)	JSLE^a^ (n = 88)	ASLE^b^ (n = 638)	LSLE^c^ (n = 71)	*P*
Age of diagnosis (years)	32.6 ± 12.5	15.4 ± 2.2	32.0 ± 8.3	59.0 ± 6.7	< 0.001
Female (%)	737 (92.5)	79 (89.8)	593 (92.9)	65 (91.5)	0.545
**Clinical features at diagnosis**					
Malar rash (%)	527 (66.1)	67 (76.1)	422 (66.1)	38 (53.5)	0.011
Discoid rash (%)	50 (6.3)	7 (8.0)	37 (5.8)	6 (8.5)	0.538
Photosensitivity (%)	205 (25.7)	23 (26.1)	168 (26.3)	14 (19.7)	0.479
Oral ulcer (%)	156 (19.6)	17 (19.3)	126 (19.7)	13 (18.3)	0.957
Arthritis (%)	530 (66.5)	54 (61.4)	430 (67.4)	46 (64.8)	0.505
Serositis (%)	157 (19.7)	12 (13.6)	128 (20.1)	17 (23.9)	0.234
Renal disorder (%)	698 (87.6)	79 (89.8)	557 (87.3)	62 (87.3)	0.803
CNS^d^ disorder (%)	30 (3.8)	4 (4.5)	24 (3.8)	2 (2.8)	0.850
Hematologic disorder (%)	477 (59.8)	54 (61.4)	386 (60.5)	37 (52.1)	0.374
Immunologic disorder (%)	514 (64.5)	51 (58.0)	417 (65.4)	46 (64.8)	0.395
ANA^e^ positive (%)	649 (81.4)	75 (85.2)	518 (81.2)	56 (78.9)	0.557
SLEDAI-2K^f^ on admission	16.26 ± 8.168	17.43 ± 9.139	16.34 ± 8.163	14.08 ± 6.474	0.031
SDI^***g***^ on admission	114 (14.3)	12 (13.6)	88 (13.8)	14 (19.7)	0.393

^a^*JSLE* juvenile-onset SLE; ^b^*ASLE* adult-onset SLE; ^c^*LSLE* late-onset SLE; ^d^*CNS* central nervous system; ^e^*ANA* antinuclear antibody; ^f^*SLEDAI-2 K* SLE Disease Activity Index 2000; ^g^*SDI* Systemic Lupus International Collaborating Clinics/American College of Rheumatology Damage Index.

The Systemic Lupus International Collaborating Clinics/American College of Rheumatology Damage Index (SDI) evaluates the organ damage caused by SLE^15^. Although not significant, the proportion of patients with SDI ≥ 1 on initial admission tends to increase with onset age. Patients with SDI ≥ 1 made up 14.3% of the study population, indicating that organ damage already existed in over 10% of SLE patients in the early stages of the disease and treatment (Table 1).

No significant differences were identified between different age-onset groups in the 12 organ systems. Renal damage occurred most frequently, affecting 12.5% JSLE, 10.2% ASLE, and 12.7% LSLE patients. Proteinuria $\ge$ 3.5 g/24 h was the main cause of renal damage, (shown in Supplementary Table 1).

The incidence of several common comorbidities of SLE patients, including hypertension, diabetes, and Sjögren's syndrome on initial admission were listed in Table 2. Sjögren's syndrome only presented in ASLE group (*p* = 0.115). Though not significant, LSLE patients tended to suffer more frequently from hypertension compared to the total study population (5.6% vs. 2.5%, *p* = 0.078).

**Table 2:** Common comorbidities occurred in patients from each age-onset group.

Comorbidities on admission	All (n = 797)	JSLE^a^ (n = 88)	ASLE^b^ (n = 638)	LSLE^c^ (n = 71)	*P*
Hypertension (%)	20 (2.5)	0 (0.0)	16 (2.5)	4 (5.6)	0.078
Diabetes (%)	30 (3.8)	5 (5.7)	22 (3.4)	3 (4.2)	0.574
Sjögren's syndrome (%)	17 (2.1)	0 (0.0)	17 (2.7)	0 (0.0)	0.115

^a^*JSLE* juvenile-onset SLE; ^b^*ASLE* adult-onset SLE; ^c^*LSLE* late-onset SLE.

To investigate the possible relationships between age and organ involvement on initial admission, abnormalities in eight systems were evaluated separately (Table 3). Mucocutaneous (73.0%), musculoskeletal (61.2%), renal (56.5%) and hematologic (48.7%) involvements were the most common involvements. Mucocutaneous involvements were more frequently observed in JSLE patients (*p* = 0.042), while the LSLE group was correlated with a higher rate of cardiopulmonary dysfunction (*p* = 0.012). The rates of renal involvement were similar among the three groups.

**Table 3:** Organ involvement among different age-onset groups.

Organ involvement on admission	All (n = 797)	JSLE^a^ (n = 88)	ASLE^b^ (n = 638)	LSLE^c^ (n = 71)	*P*
Mucocutaneous (%)	582 (73.0)	70 (79.5)	468 (73.4)	44 (62.0)	0.042
Neuropsychiatric (%)	52 (6.5)	5 (5.7)	44 (6.9)	3 (4.2)	0.650
Musculoskeletal (%)	488 (61.2)	53 (60.2)	388 (60.8)	47 (66.2)	0.663
Cardiopulmonary (%)	186 (23.3)	10 (11.4)	155 (24.3)	21 (29.6)	0.012
Gastrointestinal (%)	169 (21.2)	23 (26.1)	134 (21.0)	12 (16.9)	0.353
Ophthalmologic (%)	7 (0.9)	0 (0.0)	6 (0.9)	1 (1.4)	0.595
Renal (%)	450 (56.5)	52 (59.1)	363 (56.9)	35 (49.3)	0.411
Hematologic (%)	388 (48.7)	39 (44.3)	310 (48.6)	39 (54.9)	0.410

^a^*JSLE* juvenile-onset SLE; ^b^*ASLE* adult-onset SLE; ^c^*LSLE* late-onset SLE.

The results of laboratory tests on initial admission were listed in Table 4. Significantly higher rate of proteinuria (54.5%, 56.3% vs. 36.6%, *p* = 0.007) were present in younger-onset patients. In line with such results, haematuria rate was higher in JSLE and ASLE patients compared to LSLE (43.2%, 42.0% vs. 28.2%, *p* = 0.072), as well as the rate of increased serum creatinine (13.6%, 13.5% vs. 4.2%, *p* = 0.081), albeit not significant. These data indicated that the younger patients are possibly more susceptible to renal dysfunction. Also, the hypoalbuminemia rate was significantly increased in ASLE patients (59.9% vs. 57.3%, *p* = 0.015), possibly partially caused by renal dysfunction. However, decrease in eGFR did not follow the same pattern (6.8%, 9.6% vs. 7.0%, *p* = 0.582). Antibody profiles were not differently distributed, with ANA presented in 88.8% of the patients enrolled, and the positive rates of anti-dsDNA and anti-Sm antibodies were 51.6% and 33.3%, respectively. Incidence of decreased complement C3 (75.0% vs. 69.3%, *p* = 0.209) and C4 (58.2% vs. 68.2%, *p* = 0.132) together with anti-dsDNA positivity (53.4 vs. 51.6, *p* = 0.503) showed an elevated tendency in JSLE group and tend to decrease with age.

**Table 4:** Laboratory findings among different age-onset groups.

Lab results on admission	All (n = 797)	JSLE^a^ (n = 88)	ASLE^b^ (n = 638)	LSLE^c^ (n = 71)	*P*
Leukopenia (%)	389 (48.8)	34 (38.6)	318 (49.8)	37 (52.1)	0.121
Erythropenia (%)	330 (41.4)	33 (37.5)	267 (41.8)	30 (42.3)	0.731
Thrombocytopenia (%)	231 (29.0)	25 (28.4)	180 (28.2)	26 (36.6)	0.331
Anaemia (%)	509 (63.9)	57 (64.8)	410 (64.3)	42 (59.2)	0.685
Proteinuria (%)	433 (54.3)	48 (54.5)	359 (56.3)	26 (36.6)	0.007
Haematuria (%)	326 (40.9)	38 (43.2)	268 (42.0)	20 (28.2)	0.072
Increased ALT^d^ (%)	124 (15.6)	19 (21.6)	98 (15.4)	7 (9.9)	0.122
Increased AST^e^ (%)	138 (17.3)	17 (19.3)	112 (17.6)	9 (12.7)	0.512
Hypoalbuminemia (%)	457 (57.3)	41 (46.6)	382 (59.9)	34 (47.9)	0.015
Increased serum creatinine (%)	101 (12.7)	12 (13.6)	86 (13.5)	3 (4.2)	0.081
Increased BUN^f^ (%)	184 (23.1)	22 (25.0)	146 (22.9)	16 (22.5)	0.901
Decreased eGFR^g^ (%)	72 (9.0)	6 (6.8)	61 (9.6)	5 (7.0)	0.582
Increased ESR^h^ (%)	592 (74.3)	60 (68.2)	476 (74.6)	56 (78.9)	0.282
Positive ANA ^i^ (%)	708 (88.8)	78 (88.6)	566 (88.7)	64 (90.1)	0.935
Positive anti-dsDNA^j^(%)	411 (51.6)	47 (53.4)	332 (52.0)	32 (45.1)	0.503
Positive anti-Sm^k^ (%)	256 (32.1)	35 (39.8)	202 (31.7)	19 (26.8)	0.186
Decreased complement C3 (%)	552 (69.3)	66 (75.0)	442 (69.3)	44 (62.0)	0.209
Decreased complement C4 (%)	464 (58.2)	60 (68.2)	363 (56.9)	41 (57.7)	0.132

^a^*JSLE* juvenile-onset SLE; ^b^*ASLE* adult-onset SLE; ^c^*LSLE* late-onset SLE; ^d^*ALT* alanine aminotransferase; ^e^*AST* aspartate aminotransferase; ^f^*BUN* blood urea nitrogen; ^g^*eGFR* estimated glomerular filtration rate; ^h^*ESR* erythrocyte sedimentation rate; ^i^*ANA*, anti-nuclear antibody; ^j^*anti-dsDNA* anti-double-stranded deoxyribonucleic acid antibody; ^k^*anti-Sm* anti-Smith antibody.

## Discussion

Based on the SLE database of Jiangsu province, we carried out this study to evaluate clinical manifestations and laboratory findings among juvenile, adult, and late-onset patients, respectively. In our study population, SLEDAI-2 K decreased significantly with onset age, while SDI showed an ascending trend. More specifically, younger-onset patients are more susceptible to mucocutaneous symptoms and proteinuria, while LSLE patients tend to suffer cardiopulmonary dysfunction, notably serositis and interstitial lung disease. Renal damage was the main cause of organ damage in all age-onset groups. Our findings indicate that SLE patients with different onset ages may prone to different manifestations. As the heterogeneity of SLE requires highly individualized treatment in clinical practice^16^, these results may guide treatment decisions when facing different age-onset patients.

Clinical variations among SLE patients at different age-onset have long been recognized by rheumatologists. For example, in a multi-ethnic (69% Caucasians) study from Canada comparing clinical features in 828 JSLE and ASLE patients, neurologic disorder rate and anti-cardiolipin antibody positivity were more prevalent in JSLE^12^. In 719 JSLE and ASLE patients in Turkey, higher anti-dsDNA antibody positivity was found in JSLE. Mucocutaneous, renal, neuropsychiatric, and hematologic symptoms in JSLE were also more frequent^17^. In a Portugal cohort of 267 SLE patients, renal, hematologic, and neurologic involvements were identified to be significantly higher in JSLE than in ASLE and LSLE, and SLEDAI-2 K was found to be significantly higher^11^. In Egypt, authors identified in 575 SLE patients from three age-onset groups that except for a higher comorbidity rate, LSLE tended to be milder, with SLEDAI-2 K and SDI scores significantly lower than JSLE and ASLE. Among 201 SLE patients in Korea, anaemia, thrombocytopenia, oral ulcers, renal involvement, etc. were more common in JSLE among three age-onset groups, and SLEDAI-2 K was also significantly higher^14^. Some similar findings have arisen from these studies, like higher disease activity in JSLE patients. However, the sample sizes of researches comparing three age-onset SLE groups were relatively small, and studies from China are lacking. More data is needed to reach a concrete conclusion.

In the present study, among all patients enrolled, renal disorder, arthritis, and anaemia were the most prevalent clinical manifestations at diagnosis. Immunological indices demonstrated ANA positivity existed in nearly 90% of the patients, supporting the newly updated ACR-EULAR classification criteria requiring positive ANA at any time as an entry criterion^18^. Decreased complement or positive anti-dsDNA were both observed in more than half of the patients, the frequencies higher than in other Chinese studies, for example, in the CSTAR cohort, where anti-dsDNA positivity was around 30%^19^, possibly because we only enrolled hospitalized patients that tended to suffer more active disease course.

SLEDAI-2 K is an important indicator to evaluate the disease activity of SLE and can guide clinical medication. It has been widely reported that JSLE seemed to be more severe while LSLE was relatively milder^20–24^. The genetic background of JSLE patients, including STAT4 gene variant and long interspersed nuclear element-1 (LINE-1) hypomethylation, have been reported to contribute to the severity and disease activity of SLE^10^. By comparing SLEDAI-2 K among different age-onset groups, we found that SLEDAI-2 K was significantly elevated in younger-onset patients, indicating these patients with a more active disease at the early stages of SLE onset.

Complement C3 and C4 reduction, and anti-dsDNA positivity were also more commonly observed, implying disease activation. However, the rate of SDI ≥ 1 tended to increase with age, showing the possibility that elder patients, although seemed to present with a milder disease course, were not spared from irreversible systemic damage and loss of function.

Lupus nephritis affects nearly half of the SLE patients in China^25^ and has been pointed out to be associated with shortened survival^26^. Renal dysfunction was widely reported worldwide to be more prevalent among younger-onset patients^4,9,22,24,27–29^. Nephritis affects up to 80% of JSLE patients^8,29^ and is considered a characteristic clinical presentation for this age-onset group^29^. For JSLE patients, genetic factors, overproduction of inflammatory cytokines as well as imbalanced T cell phenotype all contribute to the risk and severity of lupus nephritis^8,10,30^. In our study, we observed that on initial admission, the proteinuria rate was significantly higher in younger-onset patients. The haematuria rates were also elevated, albeit not significant. Meanwhile, serum creatinine elevation was less commonly found among LSLE patients. And hypoalbuminemia occurrence was also significantly higher in ASLE, supporting that renal abnormalities may be more prevalent and severe among younger-onset patients. We also demonstrated based on SDI that renal damage was the most prevalent organ damage, with mass proteinuria (> 3.5 g/24 h) being the main cause, which is also slightly higher in JSLE (shown in Supplementary Table 1).

Malar rash, the most typical clinical manifestation of SLE, was more commonly found in younger-onset patients in our study population, in agreement with several recently published researches carried out in different races^4,9,17,31^. Other forms of mucocutaneous lesions, such as discoid rash, photosensitivity, and oral ulcer all followed the same trend, occurring more frequently in JSLE and ASLE populations. Previous studies have shown that the polymorphisms of genes involved in immune cell signalling (e.g., STAT4) and complement activation (e.g., MBL2) may be associated with mucocutaneous manifestations. These risk alleles are more commonly detected in JSLE patients, raising the possibility that genetic background may be a reason for the higher incidence of dermatological symptoms among younger-onset patients^10^.

In our study, the occurrence of cardiopulmonary involvement significantly increased in ASLE and LSLE populations, with serositis being the most prevalent in cardiopulmonary involvement and the interstitial lung disease rate dramatically elevated in LSLE (shown in Supplementary Table 2). Similar results have been demonstrated by two previous studies, showing cardiopulmonary involvement being more common in later-onset SLE^17,27^. A meta-analysis also agrees with our findings, showing serositis along with pleuritis and interstitial lung disease being more prevalent^32^. In a previous study, 50% of deaths of enrolled LSLE patients were due to cardiovascular events^33^. However, another study indicated that two years prior to SLE onset, LSLE patients began to experience more cardiovascular diseases^34^, raising the possibility that cardiopulmonary dysfunction was not necessarily caused solely by SLE itself, given that the incidence of cardiovascular disease tends to be higher among the elderly. In our study, we found that serositis was the main cause of cardiopulmonary involvement, and interstitial lung disease contributed the most to the elevation of cardiopulmonary involvement rate in our LSLE patients (shown in Supplementary Table 2), indicating that serositis and interstitial lung disease may be the main cause of cardiopulmonary dysfunction in LSLE patients.

Furthermore, haematological dysfunction in LSLE should also be paid attention to. A previous LSLE cohort study in China had found the hematologic system to be the most commonly affected, occurring in 53.8% of the LSLE patients at diagnosis^35^. Notably, more than half (52.1%) of LSLE patients in our study suffered leukopenia, indicating an increased risk of infection among these patients. Similarly, in LSLE patients, infection was noted as a major cause of death in other studies^35,36^. Other than leukopenia, thrombocytopenia tended to be more prevalent as well, possibly increasing the risk of bleeding.

Although JSLE and LSLE patients have been pointed out to show different disease patterns compared to the ASLE population respectively, studies systemically comparing the clinical characteristics among three age-onset groups were still lacking, and the cut-off ages used by such studies were inconsistent. Our work systemically compares the clinical features and laboratory findings of different age-onset SLE patients in a large Chinese population, choosing the cut-off ages in accordance with recent reviews and systemic reviews which were hopefully the most widely used definition. For the first time, we confirmed in a Chinese population that SLEDAI scores tend to be higher in younger patients. We also spotted some age-onset specific features like mucocutaneous disorder, and proteinuria in JSLE, and increased cardiopulmonary abnormalities in LSLE patients.

Our study has certain limitations. As mentioned above, only hospitalized patients were enrolled, this may cause selection bias as the disease activity of these patients might be higher. Also, the JLSE and LSLE populations were relatively smaller, which may add to our difficulty in identifying possible statistical significance. For example, while many studies reported a significantly higher prevalence of hypertension in LSLE patients^11,21,37^, although a similar trend was observed in our study, the difference did not reach significance. Also, we did not further analyse the differences in clinical features among pre-pubertal and adolescent SLE patients. It has been shown in a European population that the latter suffered more active disease and showing higher titre of ANA and anti-dsDNA^20^. We call for more efforts in discovering the underlying mechanism of age-of-onset associated differences in SLE.

Nevertheless, in this study, by presenting clinical and serological data according to age-onset, we demonstrated specific features of juvenile, adult, and late-onset SLE in a Chinese population. In conclusion, we found that patterns of clinical manifestations and laboratory findings associate with onset age. Younger-onset patients are more likely to experience more active disease and suffer higher occurrence of mucocutaneous involvement and proteinuria. Late-onset patients are more prone to cardiopulmonary involvement. These findings indicate that SLE can be divided into different clinical subtypes, and targeted clinical decisions can be made accordingly.

## Methods

### Study design and inclusion criteria

This study was approved by the Institutional Review Board of Nanjing Drum Tower Hospital (2020-093-01) and performed according to relevant guidelines and regulations. All methods used were in accordance with the Declaration of Helsinki. The research procedures were carried out according to the STROBE (The Strengthening the Reporting of Observational Studies in Epidemiology) guideline. Under the supervision of the Jiangsu Rheumatology Association, an online database (http://sys.91sqs.net/sle/Index/index.html) supported by Cinkate Corp was set up in 2010 for the collection of medical records of hospitalized patients in Jiangsu Province, China, from January 1, 1999, to December 31, 2009. Informed consent was obtained from all participants, or, if the participants were under 16, from their legal guardians.

All participants fulfilled at least 4 of the 1997 American College of Rheumatology (ACR) criteria for the classification of SLE^38^. After 575 were excluded due to incomplete data, 797 patients were enrolled. They were grouped into juvenile-onset SLE (JSLE), adult-onset SLE (ASLE), and late-onset SLE (LSLE) if first diagnosed at age < 18, $\ge$ 18, and $\le$ 50, > 50 years old, respectively. The cut-off ages were consistent with recent reviews and meta-analyses concerning juvenile and late-onset SLE patients^8,10,32^. Before the assessment, data allowing identification was removed and replaced by a specific number for each participant.

### Data collection and analysis

The data collection was completed from 1999 to 2009 on patients’ initial admission by face-to-face interview. The clinical features listed in Table 1 were based on the 1997 ACR revised criteria for the classification of systemic lupus erythematosus, and were collected at diagnosis^38^. SLEDAI-2 K and SDI scoring were performed on initial admission^15,39^.

The three most common concomitant diseases observed, including hypertension, diabetes, and Sjögren's syndrome on initial admission were recorded. Hypertension was defined as: systolic blood pressure $\le$ 140 mmHg and (or) diastolic blood pressure $\ge$ 90 mmHg at resting state without antihypertensive medications, measured by clinicians in triplicate on separate days, or blood pressure < 140/90 mmHg with current use of antihypertensive medication^40^. The American Diabetes Association criteria was used for the diagnosis of diabetes^41^. Sjögren's syndrome was diagnosed based on the European classification criteria^42^.

Systemic involvement was evaluated by experienced rheumatologists on initial admission, and patients with one of the following manifestations were recorded as having organ involvement: (1) *Mucocutaneous*: skin eruption, mucosal ulceration, cutaneous vasculitis, alopecia, digital infarcts, periungual erythema, angioedema or panniculitis; (2) *Neuropsychiatric*: headache, epilepsy, cerebral vasculitis, cerebrovascular disease, demyelination syndrome, myelopathy, aseptic meningitis, cerebellar ataxia, mononeuropathy, polyneuropathy, psychosis, acute confusional state, mood disorder (depression/mania); (3) *Musculoskeletal*: arthritis/arthralgia, myositis/myalgia; (4) *Cardiopulmonary*: serositis, myocarditis, interstitial lung disease, pulmonary arterial hypertension, pulmonary haemorrhage/vasculitis, cardiac failure, arrhythmia, valvular dysfunction; (5) *Gastrointestinal*: peritonitis, ascites, malabsorption, hepatitis/abnormal liver function, mesenteric vasculitis, protein-losing enteropathy, lupus gastroenteritis, pancreatitis; (6) *Ophthalmic*: orbital inflammation, keratitis, uveitis, episcleritis, scleritis, retinal/choroidal vaso-occlusive disease, isolated cotton-wool spots, optic neuritis; (7) *Renal*: proteinuria, haematuria, active urinary sediment, increased serum creatinine or abnormal glomerular filtration rate (GFR), hypertension (renal related), biopsy-proved lupus nephritis; (8) *Haematological*: haemolytic anaemia, leukopenia, thrombocytopenia^43^. The above definitions were based on the British Isles Lupus Assessment Group (BILAG) 2004 index^44^.

Laboratory results were collected on initial admission. Normal values of laboratory findings were as follows: Leukocytes ≥ 4 ×  10^9^/L, erythrocytes ≥ 3.5 ×  10^12^/L, platelets ≥ 100 ×  10^9^/L, haemoglobin ≥ 110 g/L (female) or 120 g/L (male), urine protein < 0.5 g/24 h or less than  2 +, lanine aminotransferase (ALT) ≤ 50 IU/L, aspartate aminotransferase (AST) ≤ 50 IU/L, serum albumin ≥ 35 g/L, serum creatinine ≤ 133 μmol/L, blood urea nitrogen (BUN) ≤ 7.5 mmol/L, estimated glomerular filtration rate (eGFR) ≥ 90 ml/min/1.73m^2^, erythrocyte sedimentation rate (ESR) ≤ 20 (female) or ≤ 15 mm/h (male), anti-nuclear antibody (ANA) ≤ 1: 40, anti-dsDNA(anti-double-stranded deoxyribonucleic acid) antibody negative, anti-Sm (anti-Smith) antibody negative, anti-cardiolipin antibody < 12 U/ml or negative, rheumatoid factor (RF) < 20 IU/ml, complement C3 ≥ 0.8 g/L, C4 ≥ 0.2 g/L. All the autoantibodies tested were IgG type and the positivity and negativity were defined based on the criteria of each hospital^43^.

### Statistics

Data were processed by SPSS (Statistical Package for the Social Sciences) version 22.0 software. Categorical variables were expressed as numbers and frequencies, and analysed by Chi‑square test. Normally distributed numeric variables were represented by mean ± standard deviation (SD) and analysed by one-way ANOVA (Brown-Forsythe test was used if the SDs are not equal). *P* values < 0.05 were considered statistically significant.

## Supplementary Information

Supplementary Information.

## Data Availability

The raw data cannot be shared at this time as other ongoing studies may further analyse the data.
