# Supplementary material for: Clinical and laboratorial outcome of different age-onset systemic lupus erythematosus patients in Jiangsu, China: a multicentre retrospective study
Source: Sci Rep. 2022 Jun 23;12:10683. doi: 10.1038/s41598-022-14840-4 (PMC9226048; doi:10.1038/s41598-022-14840-4)
Supplement: Supplementary file 1 — Supplementary Information. [file 41598_2022_14840_MOESM1_ESM.pdf]

Clinical and laboratorial outcome of different age-onset systemic lupus erythematosus patients in Jiangsu, China - a multicentre retrospective study

Lihui Wen[1], Ziyan Chen[1], Ziyi Jin[1], Wenyou Pan[2], Lin Liu[3], Min Wu[4], Fuwan Ding[5], Huaixia Hu[6], Xiang Ding[7], Hua Wei[8], Yaohong Zou[9], Xian Qian[10], Meimei Wang[11], Jian Wu[12], Juan Tao[13], Jun Tan[14], Zhanyun Da[15], Miaojia Zhang[16], Jing Li[17], Xuebing Feng[1], Jun Liang*[1], Huayong Zhang*[1], Lingyun Sun[1]

1. Department of Rheumatology and Immunology, Nanjing University Medical School Affiliated Nanjing Drum Tower Hospital, Nanjing, China
2. Department of Rheumatology, Huai'an First People's Hospital, Huai'an, China
3. Department of Rheumatology, Xuzhou Central Hospital, Xuzhou, China
4. Department of Rheumatology, Third Affiliated Hospital of Soochow University, Changzhou, China
5. Department of Endocrinology, Yancheng Third People's Hospital, Yancheng, China
6. Department of Rheumatology, Lianyungang Second People's Hospital, Lianyungang, China
7. Department of Rheumatology, Lianyungang First People's Hospital, Lianyungang, China
8. Department of Rheumatology, Northern Jiangsu People's Hospital, Yangzhou, China
9. Department of Rheumatology, Wuxi People's Hospital, Wuxi, China
10. Department of Rheumatology, Jiangsu Province Hospital of Traditional Chinese Medicine, Nanjing, China
11. Department of Rheumatology, Southeast University Zhongda Hospital, Nanjing, China
12. Department of Rheumatology, First Affiliated Hospital of Soochow

University, Suzhou, China
13. Department of Rheumatology, Wuxi Hospital of Traditional Chinese Medicine, Wuxi, China
14. Department of Rheumatology, Zhenjiang First People's Hospital, Zhenjiang, China
15. Department of Rheumatology, Affiliated Hospital of Nantong University, Nantong, China
16. Department of Rheumatology, Jiangsu Province Hospital and Nanjing Medical University First Affiliated Hospital, Nanjing, China
17. Department of Rheumatology, Affiliated Hospital of Jiangsu University, Zhenjiang, China
Correspondence to:
Huayong Zhang. Department of Rheumatology and Immunology, Affiliated Nanjing Drum Tower Hospital, Medical School of Nanjing University, 321 Zhongshan Road, Nanjing 210008, China. Email: huayong.zhang@nju.edu.cn.
Jun Liang. Department of Rheumatology and Immunology, Affiliated Nanjing Drum Tower Hospital, Medical School of Nanjing University, 321 Zhongshan Road, Nanjing 210008, China. Email: 13505193169@139.com.
Lihui Wen and Ziyan Chen contributed equally to this work.

Supplementary Table 1. Detailed organ damages on admission among different age-onset groups

Organ damage on admission	All (n=797)	JSLE[a] (n=88)	ASLE[b] (n=638)	LSLE[c] (n=71)	P
Ocular	2 (0.3)	1 (1.1)	1 (0.2)	0 (0.0)	0.359
Any cataract ever	1 (0.1)	1 (1.1)	0 (0.0)	0 (0.0)	0.199
Retinal change or optic atrophy	1 (0.1)	0 (0.0)	1 (0.2)	0 (0.0)	1.000
Neuropsychiatric	18 (2.3)	1 (1.1)	15 (2.4)	2 (2.8)	0.823
Cognitive impairment	12 (1.5)	0 (0.0)	12 (1.9)	0 (0.0)	0.242
Seizures requiring therapy for 6 months	3 (0.4)	1 (1.1)	2 (0.3)	0 (0.0)	0.488
Cerebrovascular accident ever	4 (0.5)	0 (0.0)	2 (0.3)	2 (2.8)	0.047
Cranial or peripheral neuropathy	1 (0.1)	0 (0.0)	0 (0.0)	1 (1.4)	0.089
Renal	85 (10.7)	11 (12.5)	65 (10.2)	9 (12.7)	0.703
Estimated or measured glomerular filtration rate <50%	20 (2.5)	3 (3.4)	16 (2.5)	1 (1.4)	0.772
Proteinuria ≥ 3.5 gm/24 hours	67 (8.4)	9 (10.2)	52 (8.2)	6 (8.5)	0.805
End-stage renal disease	22 (2.8)	6 (6.8)	12 (1.9)	4 (5.6)	0.016
Pulmonary	19 (2.4)	0 (0.0)	15 (2.4)	4 (5.6)	0.060
Pulmonary fibrosis	12 (1.5)	0 (0.0)	10 (1.6)	2 (2.8)	0.338
Shrinking lung	7 (0.9)	0 (0.0)	4 (0.6)	3 (4.2)	0.023
Pulmonary infarction	2 (0.3)	0 (0.0)	2 (0.3)	0 (0.0)	1.000
Cardiovascular	16 (2.0)	0 (0.0)	16 (2.5)	0 (0.0)	0.161

Valvular disease	2 (0.3)	0 (0.0)	2 (0.3)	0 (0.0)	1.000
Pericarditis for 6 months/pericardiectomy	14 (1.8)	0 (0.0)	14 (2.2)	0 (0.0)	0.162
Peripheral vascular	3 (0.4)	0 (0.0)	2 (0.3)	1 (1.4)	0.275
Minor tissue loss (pulp space)	1 (0.1)	0 (0.0)	1 (0.2)	0 (0.0)	1.000
Venous thrombosis with swelling/ulceration/ venous stasis	2 (0.3)	0 (0.0)	1 (0.2)	1 (1.4)	0.182
Gastrointestinal	3 (0.4)	0 (0.0)	3 (0.5)	0 (0.0)	1.000
Infarction or resection of bowel below duodenum, spleen, liver, or gall bladder	2 (0.3)	0 (0.0)	2 (0.3)	0 (0.0)	1.000
Mesenteric insufficiency	1 (0.1)	0 (0.0)	1 (0.2)	0 (0.0)	1.000
Musculoskeletal	20 (2.5)	1 (1.1)	16 (2.5)	3 (4.2)	0.486
Muscle atrophy or weakness	4 (0.5)	1 (1.1)	3 (0.5)	0 (0.0)	0.590
Deforming or erosive arthritis	16 (2.0)	0 (0.0)	13 (2.0)	3 (4.2)	0.158
Skin	18 (2.3)	0 (0.0)	17 (2.7)	1 (1.4)	0.209
Scarring chronic alopecia	16 (2.0)	0 (0.0)	15 (2.4)	1 (1.4)	0.291
Extensive scarring or panniculum other than scalp and pulp space	2 (0.3)	0 (0.0)	1 (0.2)	1 (1.4)	0.120
Skin ulceration (excluding thrombosis) for >6 months	2 (0.3)	0 (0.0)	2 (0.3)	0 (0.0)	1.000
Premature gonadal failure	8 (1.0)	2 (2.3)	6 (0.9)	0 (0.0)	0.289
SDI[d] ≥1 on admission (%)	114 (14.3)	12 (13.6)	88 (13.8)	14 (19.7)	0.393

[a]JSLE, juvenile-onset SLE; [b]ASLE, adult-onset SLE; [c]LSLE, late-onset SLE; [d]SDI, Systemic Lupus International Collaborating Clinics/American College of Rheumatology Damage Index.

Supplementary Table 2. Detailed cardiopulmonary involvement on admission among different age-onset groups

Cardiopulmonary involvement on admission	**All (n=797)**	**JSLE[a] (n=88)**	**ASLE[b] (n=638)**	**LSLE[c] (n=71)**	**P**
Myocarditis	13 (1.6)	1(1.1)	12 (1.9)	0(0)	0.459
Serositis	141(17.7）	8(9.1)	119(18.7)	14(19.7)	0.079
Interstitial lung disease	36(4.5)	2(2.3)	25(3.9)	9(12.7)	0.002
Others	20(2.5)	1(1.1)	17(2.7)	2(2.8)	0.681

[a]JSLE, juvenile-onset SLE; [b]ASLE, adult-onset SLE; [c]LSLE, late-onset SLE.
